# Review of the Quality and Reliability of Online Arabic Content on Diabetic Retinopathy: Infodemiological Study

**DOI:** 10.2196/70514

**Published:** 2026-01-07

**Authors:** Abdullah Ahmed Alabdrabulridha, Dalal Mahmoud Alabdulmohsen, Maryam Abdullah AlNajjar, Ibtisam Ahmed Algouf, Abdullah Mohammed Al-Omair, Omaima Muneer Alyahya, Mesa Ahmed Almahmudi, Abdulaziz Ahmed Al Taisan

**Affiliations:** 1 Department of Ophthalmology, College of Medicine, King Faisal University, P.O. 380 Ahsaa, Hofuf, 31982, Saudi Arabia, 966 564678882

**Keywords:** diabetic retinopathy, diabetes, online health information, arabic content, reliability, internet, online, quality, retinopathy, website, *JAMA*, DISCERN, health education, *Journal of the American Medical Association*

## Abstract

**Background:**

Diabetic retinopathy (DR) is a leading cause of vision loss, particularly in the Middle East. With the rise of online health information, many patients turn to the internet for knowledge about health conditions. However, the accuracy and quality of this information can be questionable, particularly in languages other than English.

**Objective:**

We sought to evaluate the quality and reliability of Arabic websites on DR to address this knowledge gap and improve patient care.

**Methods:**

The first 100 Arabic search results for DR were examined on Google, focusing on patient education websites in Arabic. Content was assessed using a 20-question model, quality was evaluated with the DISCERN instrument, and reliability was measured using the *Journal of the American Medical Association* (*JAMA*) benchmark. Two independent raters conducted evaluations, and data were analyzed with SPSS (IBM Corp). Descriptive statistics were used for website characteristics, and the first 10 Google web pages were compared to others using bivariate analysis with a significance level of *P*<.05.

**Results:**

A Google search yielded 178,000 websites, and the first 100 were examined, with 29 meeting inclusion criteria. Most were hospital or medical center sites (n=20, 69%). The DISCERN assessment showed a low mean score of 36.59(SD 9.32) out of 80 points, with most rated “poor” or “very poor.” The *JAMA* benchmarks indicated low reliability, with 62% (18/29) failing to meet any criteria.

**Conclusions:**

This study identified significant failings in the content, quality, and reliability of Arabic websites on diabetic retinopathy, highlighting the need for stronger evidence-based online resources focused on early disease prevention.

## Introduction

Diabetic retinopathy (DR) is a major microvascular complication of diabetes mellitus, and it is considered one of the primary causes of permanent vision loss in adults and older individuals across the globe [1]. According to the World Health Organization, the Eastern Mediterranean region has the highest prevalence of diabetes mellitus worldwide. Moreover, it is estimated that DR cases are reaching up to 31% in the Eastern Mediterranean region, which is considered to be higher than the other regions on the globe [2].

Since online-based medical health information has become easily accessible to the population, it facilitates the searching and understanding of disease symptoms, risk factors, and treatment choices. It has been implemented that browsing the internet for health information has become a widespread part of the daily routine of individuals of all ages [3]. Interestingly, in today’s digital age, online search engines are the first resource for almost 75% of patients for medical conditions [4]. People tend to seek medical online information due to the low costs, being less time-consuming, and anonymity; however, online literature can provide mixed results that can be true or misleading, and this can be due to multiple factors, such as the accuracy, readability, and quality of the reported information [5]. Patients are increasingly using the internet to find health-related information that could influence medical decisions; however, there is a risk of encountering commercially influenced content. Hence, these findings indicate that the online information available on DR may not offer sufficient guidance for medical purposes [6].

The quality of online-based ophthalmological diseases was evaluated by several studies. Unfortunately, only a few studies have documented the quality and reliability of the online content of DR [6]. The studies conducted so far have mainly centered on health-related material available in English, overlooking the need to evaluate the reliability and quality of online information in other languages. It must be mentioned that the main spoken language in the Middle East, which encounters a high rate of DR cases, is Arabic [7,8]. Due to the lack of highly standardized literature on online health content about DR in Arabic, this study seeks to address this gap by conducting a qualified evaluation of Arabic-language websites focusing on DR.

## Methods

### Study Aim, Design, and Setting

This cross-sectional website analysis was designed to evaluate the reliability and quality of Arabic online information about DR. Google.com, the most widely used search engine worldwide, held a 90.29% market share in Asia and a 97.19% market share in Africa as of May 2024. Arabic-speaking countries in the Middle East span both continents. For example, Saudi Arabia has a market share of 95.60% while Egypt has 97.38% [9]. The engine was used on May 1, 2024, to search for the Arabic term for DR, “اعتلال الشبكية السكري,” in incognito mode using a new account to avoid browser bias. The first 100 search results were examined, simulating a patient’s or a general reader’s search behavior.

### Inclusion and Exclusion Criteria

Websites were included if they were written primarily in Arabic and focused on providing educational content about DR for patients or the general public. Eligible websites were required to contain written text that addressed DR-specific topics, such as causes, symptoms, diagnosis, treatment options, and preventive strategies. The inclusion was limited to the first 100 search results to reflect typical patient behavior when searching for online health information.

**Figure 1.**
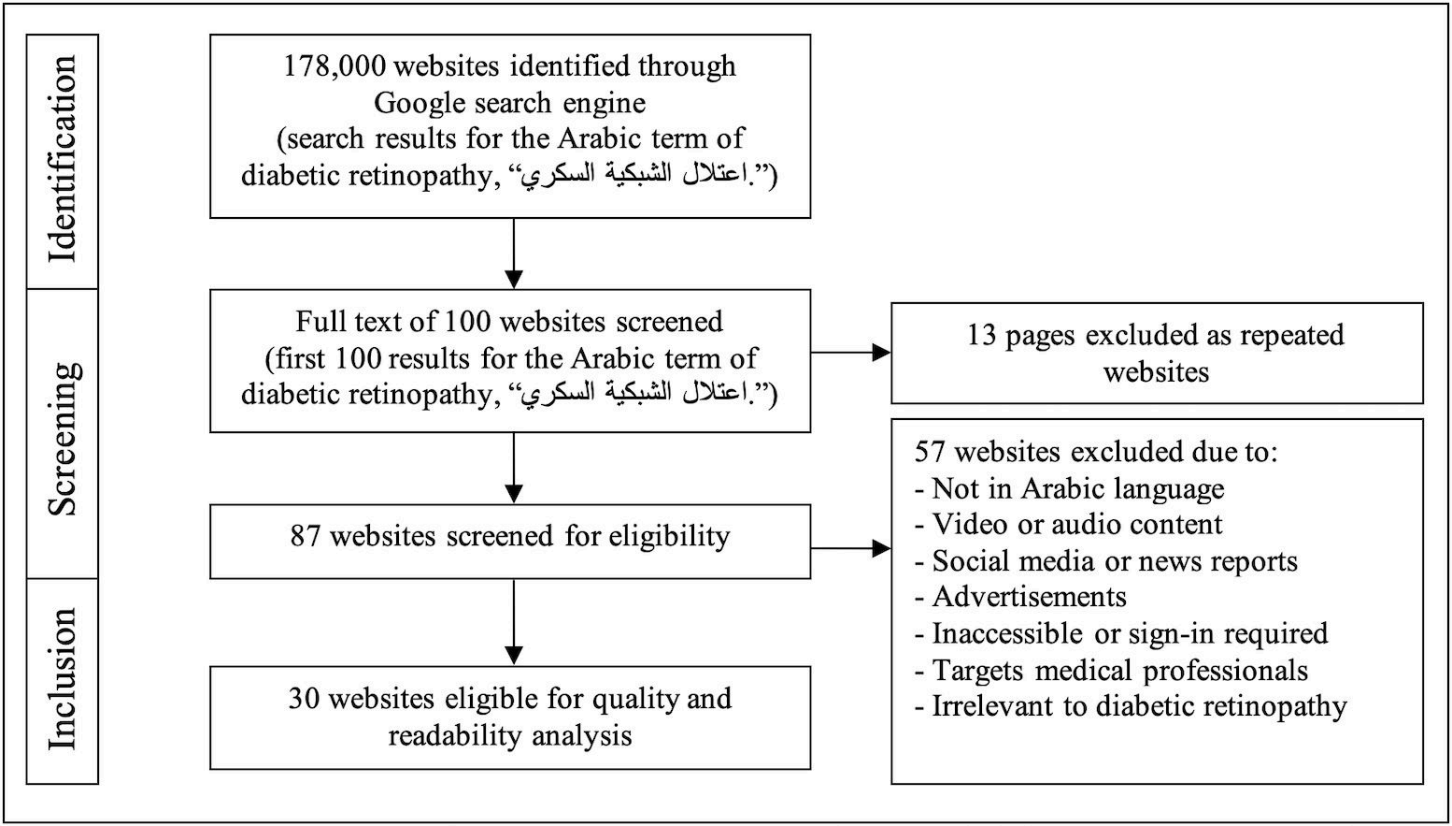
Flowchart of the search process and results.

Websites were excluded if they were not written in Arabic, targeted health care professionals (such as academic articles or clinical guidelines), or consisted solely of multimedia content like videos or audio recordings without accompanying text. In addition, websites that required login credentials, subscription access, or were otherwise inaccessible were excluded. Duplicate URLs among the first 100 search results were also removed. Finally, websites with irrelevant content, such as general diabetes pages lacking specific focus on DR, social media posts, news articles, blogs, forums, or advertisements, were excluded. The searching process is further illustrated in Figure 1.

Table 1 exhibits the list of websites eligible for evaluation. Some websites, such as Mayo Clinic, have identical URLs in different languages but automatically localize content to Arabic. Websites were classified into four main categories: hospitals and medical centers, health portals (websites dedicated to health information), commercial (websites selling a service or product), and nonprofit organizations.

**Table 1.** Websites eligible for evaluation.

Website type	Website name	URL
Nonprofit organization	Mayo Clinic [10]	https://www.mayoclinic.org
Health portals	Webteb [11]	https://www.webteb.com
Health portals	MSD Manuals [12]	https://www.msdmanuals.com
Commercial	Altibbi [13]	https://altibbi.com
Hospitals and medical centers	Cleveland Clinic Abu Dhabi [14] https://www.msdmanuals.com	https://www.clevelandclinicabudhabi.ae
Hospitals and medical centers	Institut Català De Retina [15]	https://icrcat.com
Hospitals and medical centers	Barraquer UAE Eye Hospital [16]	https://www.barraquer.com
Hospitals and medical centers	Dünyagöz [17]	https://www.dunyagoz.com
Hospitals and medical centers	Bangkok Hospital [18]	https://www.bangkokhospital.com
Hospitals and medical centers	Med Care [19]	https://www.medcare.ae
Hospitals and medical centers	Northwest Eye Surgeons [20]	https://www.nweyes.com
Hospitals and medical centers	Dr. Haifa Eye Hospital [21]	https://www.drhaifaeyehospital.com
Hospitals and medical centers	King Khaled Eye Specialist Hospital [22]	https://pep.kkesh.med.sa
Hospitals and medical centers	Royal Spanish Center [23]	https://www.royalspanishcenter.com
Hospitals and medical centers	Jgemc [24]	http://www.jgemc.com
Commercial	Vezeeta [25]	https://www.vezeeta.com
Commercial	Ilajak [26]	https://www.ilajak.com
Hospitals and medical centers	Dr Mahmoud Hassaan [27]	https://www.drmahmoud-hassaan.com
Hospitals and medical centers	Eye City Center [28]	https://www.eyecitycenter.com
Nonprofit organization	Gulf Health Council [29]	https://www.ghc.sa
Health portals	Elconsolto [30]	https://www.elconsolto.com
Hospitals and medical centers	Alkahhal [31]	https://alkahhal.com.sa
Hospitals and medical centers	Ebsaar [32]	https://ebsaar.com
Hospitals and medical centers	Smart Laser Eye Center [33]	https://www.smartlasereyecenter.com
Hospitals and medical centers	Jordan Finland Modern Hospital [34]	https://jfmhospital.com
Hospitals and medical centers	Novomed [35]	https://www.novomed.com
Commercial	Tebcan [36]	https://tebcan.com
Hospitals and medical centers	Andalusia Clinic [37]	https://www.andalusiaclinic.com
Hospitals and medical centers	Dr-Oyoun [38]	https://dr-oyoun.com
Hospitals and medical centers	Magrabi Hospital [39]	https://www.magrabi.com

### Assessment Tools

#### Content Assessment

To describe the content characteristics of the assessed websites, 20 questions were adapted from an established model by Kloosterboer et al [6] specific to DR websites. We used the model’s questions to create a scoring system ranging from 0 (no content criteria fulfilled) to 100 (all content questions covered). Of the original 26 questions, 20 were selected for their relevance to the Arabic language content and patient-oriented information. Questions that were not directly applicable were excluded.

#### Quality Assessment

The DISCERN instrument, a well-known tool for evaluating patient-targeted content quality, was also used. This questionnaire contains 16 questions with clear instructions for objective assessment, with answers ranging from 1 (no sufficient answer) to 5 (efficiently answered) [40]. The total score ranges from 16 to 80 total points. The score categories were defined by Novin et al [40], who used this tool to assess DR online information for patients, to be as follows: “Excellent (75‐63 points), Good (62‐51 points), Average (50‐39 points), Poor (38‐28 points), and Very Poor (<28 points).”

#### Reliability Assessment

The *Journal of the American Medical Association* (*JAMA*) benchmark, used to evaluate website reliability, has 4 standards, namely authorship, attribution, currency, and disclosure. Each website receives 1 point when it fulfills a standard’s criteria, with a maximum total score of 4 points. Websites scoring 3 or higher are considered highly reliable, while those scoring lower are considered low reliability [41].

### Evaluation Process

The evaluation was performed by 2 independent raters (Evaluators A and B), followed by a shared revision session to determine a final rating and resolve any disagreements from the initial evaluation. The 2 raters were senior medical students trained in the use of the DISCERN and *JAMA* benchmarks, following a structured protocol to ensure consistency and objectivity. While not board-certified ophthalmologists, the raters applied the tools according to standardized instructions that do not require clinical expertise. Raters followed the instructions of the tools to objectively determine appropriate scores for each website. The final rating was derived from the initial independent evaluations and did not substantially deviate from either rater’s original score. In all instances, the final rating either corresponded to one of the initial assessments or reflected a minor adjustment reached through consensus during the revision session.

### Data Management and Analysis

All data was organized in an Excel (Microsoft) spreadsheet and imported into IBM SPSS (version 21; IBM Corp). Descriptive statistics, such as means and SDs, were used to summarize website characteristics and evaluation scores. Normality tests were conducted to determine the data distribution (normal or nonparametric), and appropriate statistical tests were applied. Because internet users are more likely to view websites on the first page of Google (the first 10 websites) [42], these were considered to be the most viewed and therefore grouped together to be compared with the websites of other pages using both the Mann-Whitney *U* test and independent 2-tailed *t* test according to the distribution of the data. The direction of association between the DISCERN and *JAMA* scores was examined using nonparametric measures, more specifically Spearman rank correlation. A *P* value below .05 was considered statistically significant. This study was reported in accordance with the STROBE (Strengthening the Reporting of Observational Studies in Epidemiology) guidelines [43].

## Results

### Website Selection Process and Overview

Our search for the Arabic term for DR using the Google search engine yielded 178,000 websites. Examining the first 100 revealed 13 duplicates, which were excluded, and further filtering of the remaining 87 for eligibility led to the exclusion of pages in languages other than Arabic, video or audio content only, inaccessible sites requiring visitors to sign in, those targeting medical professionals only, irrelevant topics, advertisements, social media posts, and news reports. Among the 30 pages that were eligible for further analysis, one more website was excluded and removed from the dataset as it became unavailable during the analysis process.

Of the 29 websites finally included, the majority (n=20, 69%) were classified as hospitals and medical centers. Commercial pages made up 4 (13.8%) websites. Around 3 (10.3%) websites fell under the health portal classification. The remaining 2 (6.9%) websites belonged to the foundation or nonprofit organization category.

### Quality Assessment by DISCERN

The overall mean score of DISCERN for all websites was low (mean 36.59,SD 9.322), ranging from 17 to 61 points. None of the websites reached the “excellent” category (≥63 points). Only 2 of 29 (6.9%) websites scored in the “good” range (≥51 points). A total of 7 of 29 (24.1%) pages fell into the “average” category with a score ranging from 39 to 50 points. The majority of the included websites (16/29, 55.2%) had a “poor” score (38‐28 points), and the remaining 4 of 29 (13.8%) pages achieved a “very poor” score of less than 28 points.

The overall quality score (DISCERN item 16) of the identified 29 websites had an average of 2.45 (SD 0.910). The DISCERN items with the highest scores were related to the relevancy of the content, the variety of treatment options, and how each treatment works (mean 4.59, SD 0.983; mean 4.00, SD 1.000; and mean 4.10, SD 1.263, respectively; Table 2).

**Table 2.** Average scores for each DISCERN item in evaluating 29 websites, ranging from 1 to 5.

Item	Score
1. Are the aims clear?	1.38
2. Does it achieve its aims?	1.55
3. Is it relevant?	4.59
4. Is it clear what sources of information were used to compile the publication (other than the author or producer)?	1.45
5. Is it clear when the information used or reported in the publication was produced?	1.48
6. Is it balanced and unbiased?	3.62
7. Does it provide details of additional sources of support and information?	1.66
8. Does it refer to areas of uncertainty?	1.10
9. Does it describe how each treatment works?	4.10
10. Does it describe the benefits of each treatment?	2.90
11. Does it describe the risks of each treatment?	1.90
12. Does it describe what would happen if no treatment is used?	1.69
13. Does it describe how the treatment choices affect overall quality of life?	1.17
14. Is it clear that there may be more than one possible treatment choice?	4.00
15. Does it provide support for shared decision-making?	1.55
16. Based on the answers to all of the above questions, rate the overall quality of the publication as a source of information about treatment choices	2.45

Several websites scored low in relation to revealing the explicit aims of their content and their source of information (mean 1.38,SD 1.015 and mean 1.45, SD 1.055, respectively). In addition, not many pages reported the date their content was created or published (mean score 1.48, SD 0.949). Few websites mentioned the possible risks of each treatment (mean score 1.90, SD 1.345), and almost none of the websites (1/29, 3.4%) highlighted any “gray” areas or uncertainties about the outcomes of the treatments (mean score 1.10, SD 0.557). Nearly all websites (27/29, 93.1%) scored 1 point out of 5 in the question related to the effect of treatment on quality of life (mean 1.17, SD 0.658; Table 2).

### Reliability Assessment by *JAMA* Benchmarks

None of the websites obtained a score higher than 2 on the *JAMA* benchmarks tool (mean 0.45, SD 0.632), placing them all in the “low reliability” category. Over two-thirds (18/29, 62.1%) of the websites failed to meet any criteria, while the remaining websites (11/29, 37.9%) met only 1 or 2. The most common criterion met was currency, with 10 of 29 (34.5%) websites complying with it and an average score of 0.34 (SD 0.484). Authorship (mean 0.03, SD 0.186) and disclosure (mean 0.07, SD 0.258) were only displayed on 1 and 2 web pages (1/29, 3.4% and 2/29, 6.9%, respectively). No website has fulfilled the attribution benchmark. A very weak positive correlation between the DISCERN and *JAMA* scores was observed (ρ=0.130; *P*=.50).

### Content Assessment

Out of 20 questions, a single website (1/29, 3.4%) answered the most, covering 75% of the questions. On the other hand, (2/29, 6.9% of the group) websites answered the least number of questions, only covering 15%. While most websites (27/29, 93.1%) explained what DR was and how it was treated, very few mentioned the screening period for DR (8/29, 27.6%). Only 2 of 29 (6.9%) websites discussed the reversibility of vision loss caused by DR. Moreover, 5 of 29 (17.2%) of the included websites covered the surgical options to treat DR and its possible risks. The vast majority (25/29, 86.2%) lacked images of DR (Table 3).

**Table 3.** Twenty questions about the content related to diabetic retinopathy with frequencies and percentage.

Questions	Yes, n (%)		No, n (%)	
What is diabetic retinopathy?	27 (93.1)		2 (6.9)	
What are the symptoms of diabetic retinopathy?	22 (75.9)		7 (24.1)	
What is the difference between nonproliferative and proliferative diabetic retinopathy?	18 (62.1)		11 (37.9)	
How is diabetic retinopathy diagnosed?	19 (65.5)		10 (34.5)	
When should screening start?	8 (27.6)		21 (72.4)	
What are the risk factors for diabetic retinopathy?	19 (65.5)		10 (34.5)	
Can anything be done to reverse diabetic retinopathy?	9 (31)		20 (69)	
What percentage of patients become legally blind from diabetic retinopathy?	2 (6.9)		27 (93.1)	
How can vision loss be prevented?	11 (37.9)		18 (62.1)	
Is vision loss reversible?	2 (6.9)		27 (93.1)	
How is diabetic retinopathy treated?	27 (93.1)		2 (6.9)	
What is panretinal photocoagulation, and what are the complications associated with it?	8 (27.6)		21 (72.4)	
What is an anti-VEGF^a^ injection and what are the complications associated with anti-VEGF therapy?	6 (20.7)		23 (79.3)	
Are anti-VEGF injections or laser a cure or do they need to be repeated?	8 (27.6)		21 (72.4)	
What are the surgical treatments for diabetic retinopathy and what are the potential complications?	5 (17.2)		24 (82.8)	
What is tractional retinal detachment?	12 (41.4)		17 (58.6)	
What is diabetic macular edema?	12 (41.4)		17 (58.6)	
Are there any oral medications that can alter the progression of diabetic retinopathy?	1 (3.4)		28 (96.6)	
Which age group is most commonly affected by diabetic retinopathy?	2 (6.9)		27 (93.1)	
Does the source show pictures of diabetic retinopathy?	4 (13.8)		25 (86.2)	

a VEGF: vascular endothelial growth factor.

### Comparison of the Websites on the First Page and Additional Pages

The Mann-Whitney *U* test revealed no significant difference in *JAMA* scores between the first 10 websites (displayed on the first page) and those on subsequent pages (1-tailed *P*=.12). Similarly, bivariate analysis showed no significant difference in the DISCERN and content scores between the websites of the first page and other pages (*P*=.72). Detailed individual website scores for quality, reliability, and content are presented in Table 4.

**Table 4.** Included websites’ scores on quality, reliability, and content.

Website name	Quality assessment by DISCERN (16-80)	Class	Reliability assessment by *JAMA^a^* (0-4)	Class	Content score (out of 20)	Content score (out of 100)
Mayo Clinic	55	Good	2	Low reliability	15	75
Webteb	37	Poor	1	Low reliability	11	55
MSD Manuals	38	Poor	2	Low reliability	9	45
Altibbi	44	Average	0	Low reliability	9	45
Cleveland Clinic Abu Dhabi	37	Poor	0	Low reliability	10	50
Institut Català De Retina	29	Poor	1	Low reliability	4	20
Barraquer Uae Eye Hospital	25	Very poor	0	Low reliability	7	35
Dünyagöz	17	Very poor	1	Low reliability	3	15
Bangkok Hospital	35	Poor	0	Low reliability	9	45
Med Care	35	Poor	0	Low reliability	9	45
Northwest Eye Surgeons	22	Very poor	0	Low reliability	3	15
Dr. Haifa Eye Hospital	38	Poor	0	Low reliability	10	50
King Khaled Eye Specialist Hospital	36	Poor	0	Low reliability	11	55
Royal Spanish Center	41	Average	0	Low reliability	13	65
Jgemc	30	Poor	0	Low reliability	5	25
Vezeeta	27	Very poor	0	Low reliability	6	30
Ilajak	41	Average	0	Low reliability	6	30
Dr Mahmoud Hassaan	41	Average	1	Low reliability	7	35
Eye City Center	48	Average	1	Low reliability	7	35
Gulf Health Council	29	Poor	0	Low reliability	5	25
Elconsolto	61	Good	0	Low reliability	5	25
Alkahhal	41	Average	0	Low reliability	12	60
Ebsaar	28	Poor	0	Low reliability	8	40
Smart Laser Eye Center	37	Poor	1	Low reliability	8	40
Jordan Finland Hospital	34	Poor	1	Low reliability	8	40
Novomed	35	Poor	1	Low reliability	5	25
Tebcan	37	Poor	1	Low reliability	5	25
Andalusia Clinic	34	Poor	0	Low reliability	6	30
Dr-Oyoun	49	Average	0	Low reliability	6	30

a *JAMA*: *Journal of the American Medical Association.*

## Discussion

### Principal Findings

Our study assessed the reliability and quality of the contents of 29 Arabic websites covering DR based on DISCERN and *JAMA* benchmarks. Using the DISCERN tool to evaluate the quality of each website has shown an overall poor quality of all websites evaluated, with an average score of 36.59 (SD 9.322). None of the 29 websites assessed achieved “excellent,” with the majority of the websites falling under the “poor” (16/29, 55.2%) and “very poor” (4/29, 13.8%) categories. The content of the websites seems to falter in the first section of the DISCERN instrument focusing on the trustworthiness of the content presented (questions 1‐8), which reflects the unreliability of the websites offering information on DR in Arabic. In addition, comprehensive reporting of treatment options with their associated benefits and risks was inconsistent between the websites, compromising the overall quality of the content presented. Our study found that the average score of the overall quality on the DISCERN tool is 2.45 (SD 0.9), which falls under the category of having “potentially important but not serious shortcomings.” This outcome is similar to the results found by Novin et al [40], who evaluated the information about DR on US-based online websites and observed a mean score of 2.09 (SD 0.594), and similar to our results, no website was classified as “excellent” in their analysis. Moreover, both studies observed a deficiency in directing the readers to discuss their conditions with their physicians. While a perfect score of 5 signifies the highest quality, none of the analyzed websites achieved this benchmark.

Given the consistently poor quality and reliability scores across websites, these findings may be explained by several underlying factors. Hypotheses include (1) the absence of standardized quality controls in Arabic web content, (2) limited contributions from medical institutions in developing and maintaining patient education resources, and (3) a general lack of regulatory oversight governing the accuracy and reliability of health information published online. Such systemic gaps likely underlie the consistently low quality and reliability scores observed in our assessment.

Similar to the DISCERN assessment of the websites, assessing the 29 websites included with the *JAMA* benchmarks tool has revealed that all the websites are of low reliability. None of the websites evaluated obtained a score higher than 2, with an average score of 0.45 (SD 0.632). Comparing our results to the study done on dry eye disease websites, their assessment using *JAMA* has found that all the websites failed to meet half the benchmarks as well, with an average of 1.9 (SD 0.1) [44]. The low average in our study is due to more than two-thirds (18/29, 62.1%) of the websites assessed failing to meet any of the 4 *JAMA* benchmark criteria for a reliable website. A study conducted by Kloosterboer et al [6] on assessing online information regarding DR has also found that none of the websites evaluated has achieved all 4 *JAMA* benchmarks.

Upon evaluating each website’s content, it was observed that no website has covered all questions, with the highest-scoring website only fulfilling 75% of the questions (n=15). While most websites included information on what DR is and how it is treated, many of them (20/29, 69%) lacked information about when screening should begin. The study by Novin et al [6] also noted this, pointing out that there was insufficient content on the DR screening intervals for type 1 and type 2 diabetes. A substantial number of the evaluated websites did not thoroughly address every therapeutic choice with its correlated risks and benefits. This was also a common finding in another study assessing DR-related internet resources for patients, where most websites scored in the lower range in questions related to treatment options.

The study found the average score for the balance and unbiasedness item of the DISCERN instrument to be 3.62 (SD 1.24) for Arabic websites covering DR. This rating may be attributed to the inclusion of commercial websites in the evaluation, despite excluding those explicitly labeled as “advertisement” or “ad” in Google search results. These commercial sites, while offering health services or products, also provide health educational content. The presence of commercial links and advertisements can hinder users’ ability to locate reliable information, contributing to a “misinfodemic” and making it challenging for patients to access trustworthy health resources [45].

We believe that the gap found in the quality of content reported on all the assessed websites reflects the current health care practices in the region, where collaborative decision-making and thorough explanation of treatment risks and benefits may not always be given priority. A systematic literature review of patient-centered care (PCC) in the Middle East concluded that while there is support for adopting PCC in the Middle East and North African region, its implementation is still limited [46]. Webair identified barriers to adopting a PCC approach at multiple levels, mostly related to communication, suggesting a preference for a physician-driven approach that may not place as much emphasis on discussing treatment alternatives with patients [47].

Our study has its own set of limitations that require addressing for future investigation and research. First, Google was the primary search engine used in this study. Although it is a well-renowned and widely used search engine, the use of other search engines could provide more websites that were not taken into consideration by relying solely on Google [9]. The second limitation is the exclusive focus on written Arabic-language content. Video-based or multimedia educational resources, which are increasingly used by patients, were excluded from this analysis. Evaluating such content could provide further insights into the quality of health information consumed by the general population through popular platforms, such as WhatsApp (Meta Platforms Inc), YouTube (Google), or TikTok (ByteDance). While the quality of the content was assessed thoroughly in our study, assessing the readability of the websites included could provide insight into whether the information provided can be adequately comprehended by patients or not. Further studies should be performed in this area to assess whether websites are within the acceptable reading levels of online educational materials.

The study shows the need to improve the quality and reliability of Arabic-language online content on DR. Content creators should follow best practices in health communication, including clear authorship, proper source attribution, transparency regarding sponsorship, and regular content updates. Information should also be patient-centered, discussing treatment options, associated risks, and preventive measures like regular screening.

At the policy level, national guidelines are needed to ensure the quality of Arabic online health information. Policymakers may consider implementing standardized accreditation systems like the Health on the Net Code of Conduct, adapted to the region’s linguistic and cultural context, to combat misinformation and enhance public health literacy.

### Conclusion

Our study’s findings disclose that Arabic-language websites providing information on DR treatment are significantly deficient in quality, reliability, and content. The DISCERN assessment tool displayed a “poor” score for most of the analyzed websites (16/29, 55.2%). In addition, according to the *JAMA* benchmark criteria, all of the websites showed low reliability, and none of them met the attribution benchmark. These findings highlight the necessity of enhancing the Arabic-language websites discussing DR treatment due to the high prevalence of this condition among patients in the Middle East and the insufficient high-quality online resources available. Middle Eastern health care organizations should collaborate to provide reliable, evidence-based online resources addressing this serious condition. While most websites discuss the treatment of DR, more information is needed on the associated risks and benefits, as well as a stronger focus on joint decision-making. In addition, Arabic websites should encourage screening and preventive measures to enhance patient outcomes.

## Abbreviations

DR: diabetic retinopathy
*JAMA*: *Journal of American Medical Association*
PCC: patient-centered care
STROBE: Strengthening the Reporting of Observational Studies in Epidemiology
